# Circulating angiogenic factors are related to the severity of gestational hypertension and preeclampsia, and their adverse outcomes

**DOI:** 10.1097/MD.0000000000006005

**Published:** 2017-01-27

**Authors:** Alfredo Leaños-Miranda, Francisco Méndez-Aguilar, Karla Leticia Ramírez-Valenzuela, Marilyn Serrano-Rodríguez, Guadalupe Berumen-Lechuga, Carlos José Molina-Pérez, Irma Isordia-Salas, Inova Campos-Galicia

**Affiliations:** a Medical Research Unit in Reproductive Medicine, UMAE-Hospital de Ginecología y Obstetricia “Luis Castelazo Ayala,” Instituto Mexicano del Seguro Social; b Division of Maternal-Fetal Medicine, UMAE-Hospital de Ginecología y Obstetricia “Luis Castelazo Ayala,” Instituto Mexicano del Seguro Social; c Department of Obstetrics and Gynecology, Hospital General Regional No. 251 Metepec, Instituto Mexicano del Seguro Social; d Research Unit in Thrombosis, Hemostasis, and Atherogenesis, Hospital Gabriel Mancera, Instituto Mexicano del Seguro Social, México, D.F., México.

**Keywords:** adverse perinatal and maternal outcomes, angiogenic factors, hypertensive disorders of pregnancy

## Abstract

Gestational hypertension (GH) and preeclampsia (PE) are characterized by an imbalance in angiogenic factors. However, the relationship among these factors with the severity of hypertensive disorders of pregnancy (HDP) and adverse outcomes are not fully elucidated. We examined whether these biomarkers are related with the severity of HDP and adverse outcomes.

Using a cross-sectional design, serum concentrations of placental growth factor (PlGF), soluble fms-like tyrosine kinase-1 (sFlt-1), and soluble endoglin were determined in 764 pregnant women: 75 healthy pregnant, 83 with mild GH (mGH), 105 with severe GH (sGH), 122 with mild PE (mPE), and 379 with severe PE (sPE).

All angiogenic factors’ concentrations were significantly different (*P* ≤ 0.041) in HDP than in healthy pregnancy. In addition, these factors were markedly different in sPE than in mPE, sGH, or mGH (*P* ≤ 0.027) and in patients with sGH that in those with mPE or mGH (*P* < 0.05). As compared to mGH and mPE, patients with sGH and sPE had higher rates of both preterm delivery at <34 weeks of gestation and small-for-gestational age infants. Moreover, patients with sPE had higher rates of adverse maternal outcomes (*P* < 0.001) when compared to patients with mGH, sGH, or mPE. In all cases, levels of sFlt-1/PlGF ratio were significantly higher in patients with sGH and sPE who had adverse perinatal and maternal outcomes than in those with sGH and sPE who did not (*P* ≤ 0.016).

Circulating concentrations of angiogenic factors appear to be suitable markers to assess the severity of GH and PE, and adverse outcomes.

## 1 Introduction

Hypertensive disorders of pregnancy (HDP) affect about 7% to 10% of all pregnancies and remain leading causes of maternal and perinatal morbidity and mortality. Gestational hypertension (GH) and preeclampsia (PE) are HDP, both disorders occurring after week 20 of pregnancy and resolve within 12 weeks after delivery. The only difference between GH and PE is the presence of significant proteinuria in the latter condition, and these disorders are classified as mild or severe depending on the systolic and/or diastolic blood pressure readings.^[1,2]^

It has been demonstrated that women who had severe GH (sGH) had increased rates of adverse perinatal outcomes (preterm delivery and small-for-gestational-age [SGA] infants) than in women who had mild GH (mGH) or mild PE (mPE), but similar to those who had severe PE (sPE).^[3,4]^ Although the cause of HDP remains unknown, accumulating evidence suggests that these disorders result from an imbalance between placental pro- and anti-angiogenic factors which damage maternal vascular endothelium, leading to the clinical manifestations of these conditions.^[5,6]^ Lower circulating concentrations of placental growth factor (PlGF) and higher concentrations of soluble vascular endothelial growth factor receptor-1 (also referred as soluble fms-like tyrosine kinase-1 [sFlt-1]), sFlt-1/PlGF ratio, and soluble endoglina (sEng) are present at the time of diagnosis of PE, and have also been associated with increase risk to develop this condition or adverse outcomes.^[7–11]^

Although several studies have shown that serum concentrations of pro- and anti-angiogenic factors in women with GH are intermediate between those found in healthy pregnant women and women with PE, neither of these studies differentiated between mGH and sGH in their analysis.^[8,10,12–15]^ Therefore, the relationship among these angiogenic factors and the severity of GH and adverse outcomes has not been examined.

The goal of the present study was to evaluate whether circulating concentrations of pro- and anti-angiogenic factors are associated with severity of disease as well as to adverse outcomes in women with GH and PE.

## 2 Patients and methods

The study protocol was approved by the scientific and ethics committees of Instituto Mexicano del Seguro Social (approval number R-2012-785-030), and all participating subjects signed an informed consent. All women were patients admitted to the Clinic of HDP of our hospital. Study participants were at gestational age 20 wk or older and had new-onset hypertension. Hypertension was defined as systolic or diastolic blood pressure ≥140 or ≥90 mm Hg, respectively, measured twice at least 4 h apart or 1 systolic or diastolic blood pressure ≥160 or ≥110 mm Hg, respectively, treated with antihypertensive medication, and that returned to normal values within 3 months after delivery. HDP were defined according to the American College of Obstetricians and Gynecology criteria (2). GH was defined as isolated hypertension without significant proteinuria (mGH ≥140/90 mm Hg and sGH ≥160/110 mm Hg). mPE or PE without severe features was defined as hypertension and significant proteinuria (≥300 mg protein in a 24-h urine specimen or a protein to creatinine ratio ≥0.30 mg/mg in a random urine sample).^[16]^ sPE or PE with severe features was considered when either hemolysis, elevated liver enzymes, low platelet count (HELLP) syndrome, eclampsia, or PE with severe hypertension (systolic or diastolic blood pressure ≥160 or ≥110 mm Hg, respectively) was present. Other parameters included, even in absence of significant proteinuria were new-onset cerebral or visual disturbances, abnormal liver enzymes levels (to twice normal concentration), or pulmonary edema. The adverse maternal outcomes included maternal mortality and any of the following serious maternal morbidities: hepatic hematoma or rupture (confirmed by ultrasound or laparotomy), pulmonary edema (clinical diagnosis and with radiographic confirmation), need for positive inotropic support, intubation (other than solely for caesarean section), acute renal failure (creatinine ≥198 μmol/L), and placental abruption (clinical or pathological). The adverse neonatal outcomes studied included preterm delivery, the death (stillbirths [defined as death of a fetus] and neonatal death [defined as death a newborn until hospital discharge]) and SGA infant, defined as an infant whose birth weight was below the 10th percentile.

A random urine sample was collected and a venous blood sample was simultaneously drawn. Samples were centrifuged, and the resulting sera and sediment-free urine specimens were aliquoted and stored at −80°C until assayed. All samples were collected before delivery. Clinical and delivery outcomes were recorded, and in those patients with GH, another random urine sample was collected at time of delivery to classify the patients with HDP. None of the women studied had preexisting hypertension or diabetes mellitus, renal diseases, connective tissue disorders, or other high-risk obstetric condition.

Serum levels of sFlt-1, PlGF, and sEng were determined in duplicate by ELISA (R&D Systems, Minneapolis, MN) following the manufacturer's instructions. The sFlt-1/PGF ratio was calculated from the corresponding sFlt-1 and PlGF values. The intra- and inter-assay coefficients of variations were 5.7% and 6.2%, respectively. Urinary protein and creatinine were measured as previously described.^[16]^

### 2.1 Statistical analysis

Differences between continuous variables were determined by the unpaired Student *t* test (or the Mann–Whitney *U* test for non-normally distributed variables). Differences between categorical variables were determined by the Chi-squared test with Yates continuity correction or the Fisher exact test for small samples (or the Mantel–Haenszel χ^2^ test with linear tendency for variables with >2 categories). Differences among ≥3 continuous variables were determined by 1-way analysis of variance (ANOVA) followed by post hoc procedures (Scheffe test) or by the Kruskal–Wallis 1-way test followed by the Mann–Whitney *U* test for non-normally distributed variables. Multiple logistical regression models were additionally used to adjust the relationship among levels of angiogenic factors and the adverse maternal and perinatal outcomes studied. A 2-tailed *P* < 0.05 was considered statistically significant.

## 3 Results

### 3.1 General description of the population studied

**Figure 1:**
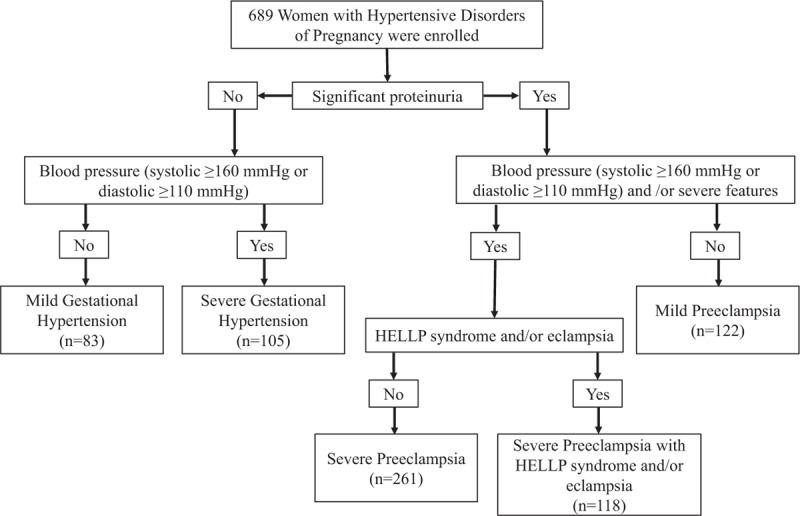
Flow chart showing patient selection for hypertensive disorders of pregnancy.

A total of 689 consecutive women with HDP were included in the study. Patients were classified into 5 groups according to clinical severity and delivery outcomes (Fig. 1): Group I, patients with mGH (n = 83); Group II, sGH (n = 105); Group III, mPE (n = 122); Group IV, sPE, but without HELLP syndrome or eclampsia (n = 261); and Group V, sPE with HELLP syndrome and/or eclampsia (n = 118 [89 patients with HELLP syndrome, 17 patients with eclampsia, and 12 women with both conditions]). In addition, 75 healthy pregnant women who remained normotensive throughout their pregnancies, and who delivered a healthy term infant (≥38 weeks), from whom serum samples were collected 3 times at 28, 32, and 36 weeks of gestation were included as control group. The demographic, clinical, and obstetric characteristics of the patients are shown in Table 1. There were no significant differences among study groups in terms of maternal age, gravidity, nulliparity, prior miscarriage, history of PE, or rate of smoking during pregnancy. Patients with GH or mPE were more likely to have higher body mass index measurements compared with women with sPE (groups IV and V). Compared with mGH and mPE women, patients with sGH and sPE (groups IV and V) had higher blood pressures (both systolic and diastolic), lower gestational age (both at enrollment and at delivery) as well as lower time elapsed between enrollment and delivery, delivered infants with lower birth weights, and had a greater proportion of preterm delivery (both at <37 and 34 weeks of gestation) and SGA infants. The groups IV and V had a greater proportion of stillbirths or early neonatal death compared with patients with mGH, sGH, and mPE.

**Table 1:**
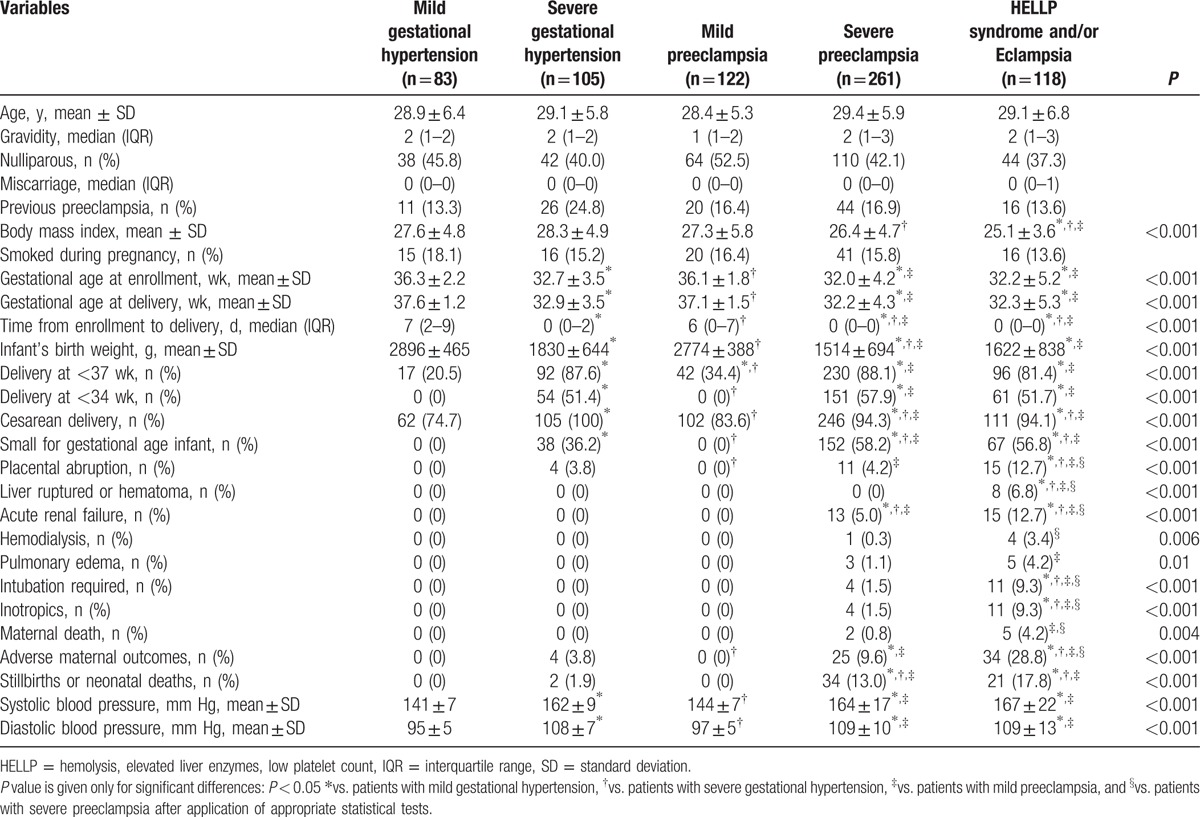
Clinical and demographic characteristics of pregnant women with hypertensive disorders of pregnancy.

Sixty-three patients had ≥1 adverse maternal outcomes, and all were in groups II, IV, and V. The occurrence of the combined adverse maternal outcomes and the various individual adverse outcomes, including placental abruption, hepatic hematoma, or rupture, acute renal failure, need for inotropic drug support, as well as endotracheal intubation, and maternal death were significantly higher in groups IV and V than in the other studied groups. Overall, patients who had sGH had more adverse perinatal outcomes than patients who had mGH or mPE, but lower than patients who had sPE (groups IV and V). By contrast, there were no statistically significant differences in perinatal outcomes between patients with mGH and mPE, except for preterm delivery at <37 weeks.

Among the 54 patients with sGH who delivered at <34 weeks of gestation, 51 (94.4%) of deliveries were indicated by severe hypertension and the remaining cases by fetal distress.

Clinical laboratory results are shown in Table 2. The final clinical diagnosis was supported by determining the protein to creatinine ratio in random urine samples (16). All women with PE (groups III–V) had significant proteinuria and had also greater degrees of proteinuria compared with mGH or sGH. In addition, proteins to creatinine ratios were significantly higher in sPE (groups IV and V) than in mPE. Compared with other groups, patients with sPE with HELLP syndrome and/or eclampsia had higher serum levels of liver enzymes and lactate dehydrogenase, as well as lower platelet counts. In addition, patients with sPE with HELLP syndrome and/or eclampsia had higher serum creatinine and uric acid levels, compared with mGH, sGH, or mPE. Serum creatinine levels were significantly higher in patients with sPE (group IV) than in mGH and sGH. Serum uric acid levels were significantly higher in patients with sPE (group IV) than in patients with mGH.

**Table 2:**
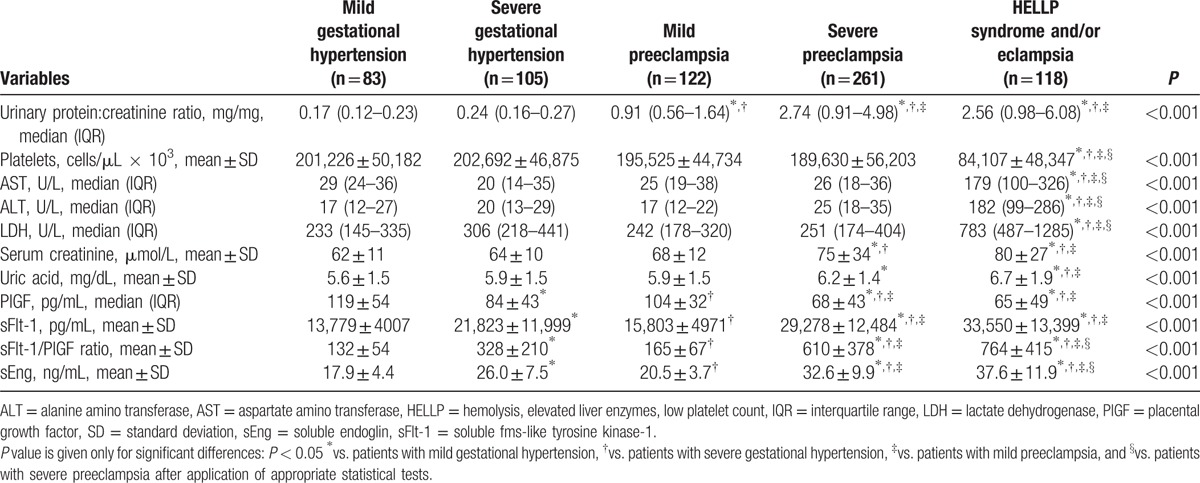
Clinical laboratory results of pregnant women with hypertensive disorders of pregnancy.

### 3.2 Relationship between circulating levels of angiogenic factors with severity of HDP

**Figure 2:**
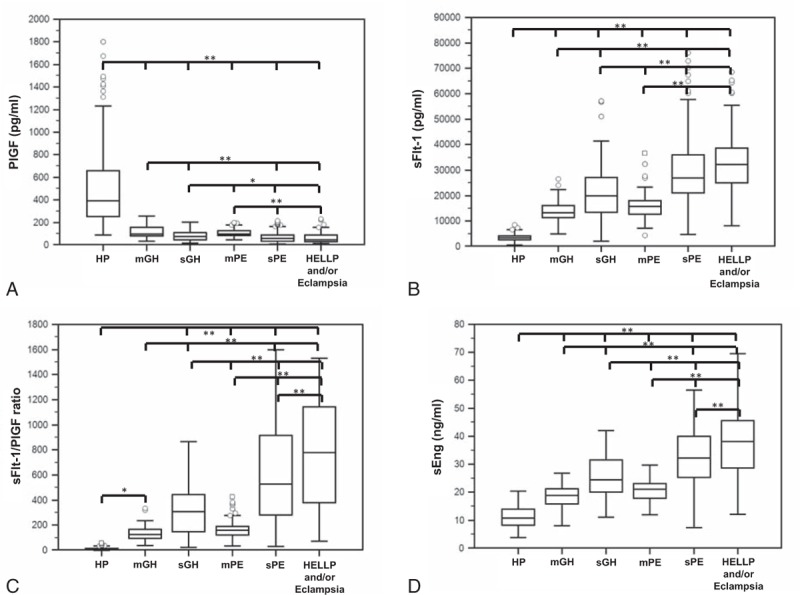
Box-and-whisker plot comparing serum concentrations of placental growth factor (PlGF; A), soluble fms-like tyrosine kinase-1 (sFlt-1; B), sFlt-1/PlGF ratio (C), and soluble endoglin (sEng; D) in women with healthy pregnancies (HP) or with hypertensive disorders of pregnancy. Boxes represent interquartile range in which the horizontal line represents the median; the top and bottom horizontal lines of the box are the 75th and 25th percentiles of the data for each group and whisker at top and bottom are the 90th and 10th percentiles. Open circles represent extreme values. Significant differences are marked with asterisks (^∗^*P* ≤ 0.041, ^∗∗^*P* ≤ 0.001). HELLP = hemolysis, elevated liver enzymes, low platelet count syndrome, mGH = mild gestational hypertension, mPE = mild preeclampsia, sGH = severe gestational hypertension, sPE = severe preeclampsia.

Serum concentrations of angiogenic factors are shown in Table 2 and Fig. 2. Compared with healthy pregnant, patients with HDP had lower serum PlGF levels and higher sFlt-1 and sEng levels, as well as sFlt-1/PlGF ratios (*P*≤0.01) (Fig. 2). Serum PlGF levels were significantly lower and sFlt-1 levels significantly higher in groups IV and V than in groups I, II, and III (*P* < 0.001) and in group II than in groups I and III. Serum concentrations of sEng and sFlt-1/PlGF ratios were significantly higher in group V than in groups I, II, III, and IV (*P* < 0.001); in group IV than in groups I, II, and III (*P* < 0.001); and in group II than in groups I and III. Although serum levels of PlGF tended to be lower and sFlt-1, sEng, and sFlt-1/PlGF ratios to be higher in patients in groups III than in group I, the differences were not statistically significant.

### 3.3 Relationship between circulating levels of angiogenic factors with adverse outcomes in sGH and sPE

Logistical regression analyses were used to determine whether serum angiogenic factors’ concentrations are associated with adverse outcomes in patients with sGH or sPE (groups IV and V) (Table 3). In sGH patients who delivered at <34 weeks’ gestation, serum PlGF concentrations were significantly lower, and sFlt-1 concentrations and sFlt-1/PlGF ratios were significantly higher than in those who delivered after 34 weeks (*P* ≤ 0.008), whereas serum sEng levels were not significantly different between sGH patients who delivered before or after 34 weeks’ gestation. In addition, in sGH patients with an SGA infant, serum PlGF concentrations were significantly lower, and sEng concentrations and sFlt-1/PlGF ratios were significantly higher than in those without an SGA infant (*P* ≤ 0.01), whereas serum sFlt-1 levels were not significantly different between sGH patients with or without an SGA infant.

**Table 3:**
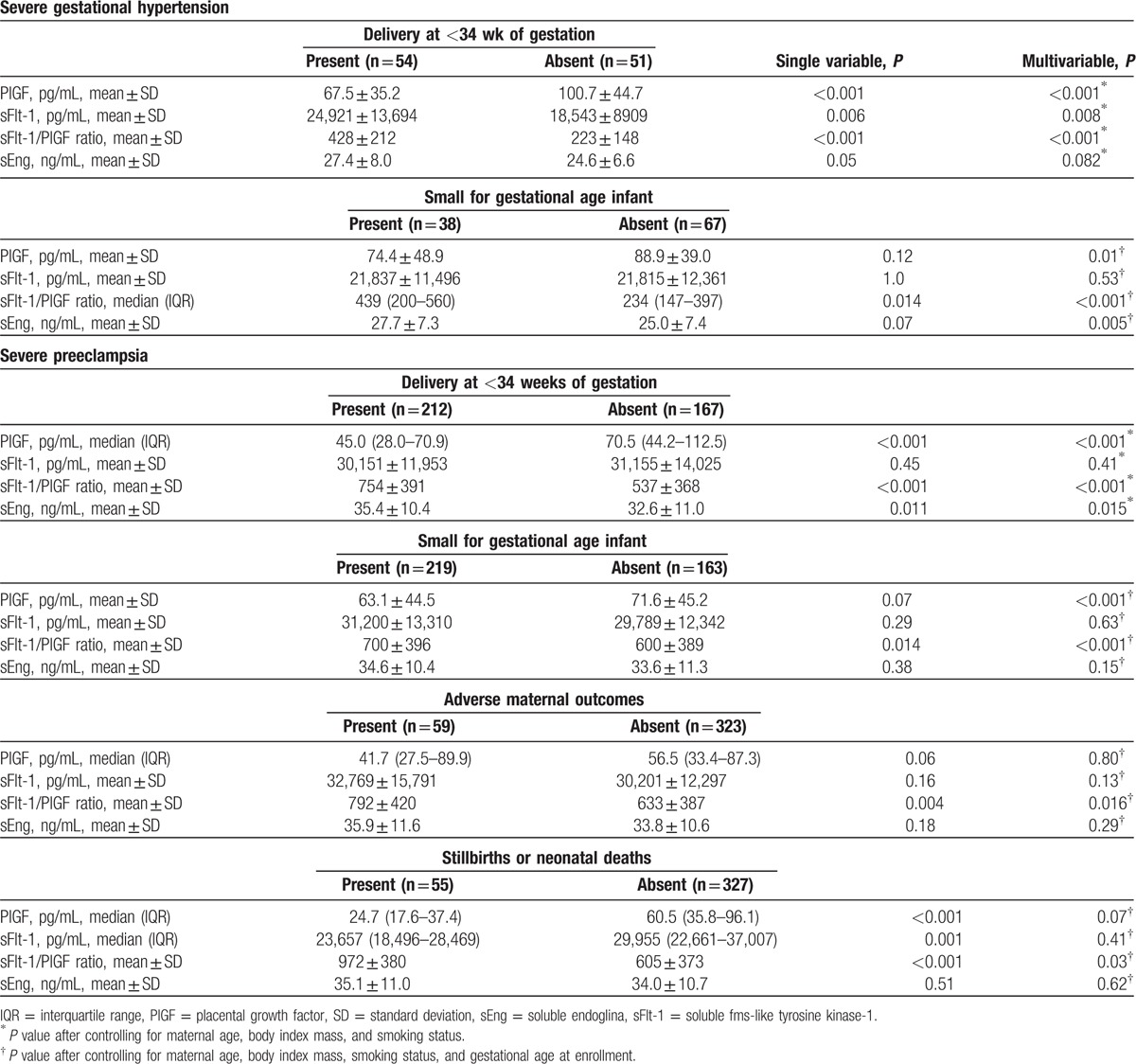
Perinatal and maternal outcomes according to serum concentrations of angiogenic factors in patients with severe gestational hypertension and severe preeclampsia.

In patients in groups IV and V who delivered at <34 weeks’ gestation, serum PlGF concentrations were significantly lower, and sEng concentrations and sFlt-1/PlGF ratios were significantly higher than in those who delivered after 34 weeks (*P* ≤ 0.015), whereas serum sFlt-1 levels were not significantly different between patients in groups IV and V who delivered before or after 34 weeks’ gestation. In patients in groups IV and V with an SGA infant, serum PlGF concentrations were significantly lower, and sFlt-1/PlGF ratios were significantly higher than in those without an SGA infant (*P* < 0.001), whereas sFlt-1 and sEng concentrations were not significantly different between patients in groups IV and V with or without an SGA infant. In addition, serum sFlt-1/PlGF ratios were significantly higher in patients in groups IV and V who developed any adverse maternal outcome or the occurrence of stillbirths or neonatal deaths than in those who did not (*P*≤0.03), whereas serum PlGF, sFlt-1, and sEng levels were not significantly different.

## 4 Discussion

The present study, involving a large number of women with HDP with a wide spectrum of disease severity and using detailed diagnostic criteria to define significant proteinuria, GH, and PE, demonstrates that alterations in circulating angiogenic factors are not only associated with the severity of PE, but they are also associated with the severity of GH. In our study, we found that serum PlGF, sFlt-1, sEng, and sFlt-1/PlGF ratio levels in patients who had GH and PE are significantly different compared with healthy pregnant women, as previously demonstrated in other studies.^[8,10,12–15]^ Furthermore, we were able to find that differences in circulating angiogenic factors were more pronounced as the severity of both GH and PE increased, suggesting that changes in serum angiogenic factors concentration effectively reflect the extent and intensity of damage to the systemic vascular endothelium that lead to severe hypertension. Also of interest was the finding that patients with mPE exhibited patterns of circulating angiogenic factors similar to those patients with mGH. In addition, the changes in serum angiogenic factors concentrations in patients with sGH were higher than in patients with mGH or mPE, but lower when compared with patients with sPE. We thus have confirmed and extended previous observations as to the relationship between alterations in circulating angiogenic factors with PE severity.^[11]^ Several previous studies have reported that changes in circulating angiogenic factors in women with GH were lesser than in those with PE.^[8,10,12–14]^ However, and in contrast to the data presented herein, neither of these studies differentiated mild from sGH or sPE. In this vein and to our knowledge, the present report represents the first that has examined and compared the circulating angiogenic factors’ concentrations in patients with GH and PE according to 2 categories, “mild” and “severe.”

Although the relationship between GH and PE remains ill defined, it is well known that GH and PE shared many clinical risk factors^[17]^ as well as an imbalance in circulating angiogenic factors^[8,10,12–15]^ (present study) and the fact that between 15% and 46% of women with GH progressed to PE,^[18–20]^ suggesting that GH and PE may be a continuous process with the same pathophysiology rather than a separate abnormal condition, even the GH has been considered as a subclinical PE.^[14]^ In this regard, it is worth mentioning that in all patients with GH in our study the clinical and laboratory findings associated with severe features of PE were absent, and the fact that the presence of significant proteinuria was ruled out, as evaluated by the protein:creatinine ratio at the time of delivery, we might argue that none of them progressed to PE.

In addition, as the changes in circulating angiogenic factors in mGH patients were almost similar to those patients with mPE, and as the changes in the concentration values of these particular angiogenic factors in patients with sGH and sPE were markedly different than in patients with mGH and mPE, suggesting that the degree of the imbalance of circulating angiogenic factors may be associated with the degree of elevation of blood pressure, but not with the levels of proteinuria. We speculate that other factors may be necessary to induce proteinuria.

The frequency of adverse maternal and neonatal outcomes was higher in patients with sPE (with or without HELLP syndrome or eclampsia) than in those with mGH, sGH, or mPE. Moreover, patients with sGH had higher rate of adverse neonatal outcomes when compared with patients who had mGH or mPE. These findings support and extended previous observations,^[3,4]^ indicating that patients with hypertensive disorder of pregnancy who developed severe hypertension had significantly higher rates of neonatal and maternal complications as compared with patients who had mGH or mPE. In this vein, a previous study has demonstrated that severe hypertension, but no proteinuria was significantly associated with adverse outcomes.^[4]^ Although it has been reported that women with sGH had outcomes similar to those with sPE^[3,4]^; in our study, we found that adverse maternal and neonatal outcomes in patients with sGH were lower to compared those with sPE. This discrepancy may stem from differences in the gestational age at diagnosis of disease, the severity of PE, and/or the limited sample size, leading to a low power of study results.

In patients with sGH and sPE who delivered at <34 weeks’ gestation or who had an SGA infant, serum PlGF concentrations were significantly lower than those who delivered after 34 weeks or without an SGA infant. These data are similar to those yielded by a previous studies in patients with PE^[7,14]^ and GH.^[14]^ However, and in contrast to the data presented herein, neither of these studies differentiated mild from sPE or sGH.

In patients with sGH and sPE, we also showed that the sFlt-1/PlGF ratio was more tightly associated with the occurrence of adverse maternal and neonatal outcomes than any of individual factors. These data are consistent with previous studies demonstrating that measurement of this ratio is a better predictor of PE and adverse outcomes of pregnancy than either measure alone.^[8–11]^ Because of the relatively small sample size, we were unable to examine in detail the various adverse outcomes either combined or individually.

In conclusion, our results demonstrate that circulating concentrations of PlGF, sFlt-1, sFlt-1/PlGF ratio, and sEng are closely associated with the severity of both GH and PE. Adverse perinatal outcomes, specifically to have preterm delivery at <34 weeks’ gestation or having an SGA infant are significantly higher in patients with sGH when compared with those with mGH or mPE, but lower to those with sPE. In particular, measurement of the sFlt-1/PlGF ratio has potential relevance as a prognostic biomarker for adverse perinatal and maternal outcomes in patients with GH similar to those patients with PE. Further prospective longitudinal studies are still needed to assess the potential role of the angiogenic factors in the diagnostic and management of HDP.
